# Directing Axonal Growth: A Review on the Fabrication of Fibrous Scaffolds That Promotes the Orientation of Axons

**DOI:** 10.3390/gels8010025

**Published:** 2021-12-28

**Authors:** Devindraan Sirkkunan, Belinda Pingguan-Murphy, Farina Muhamad

**Affiliations:** Department of Biomedical Engineering, Faculty of Engineering, Universiti Malaya, Kuala Lumpur 50603, Malaysia; ezvn2066@gmail.com (D.S.); bpingguan@gmail.com (B.P.-M.)

**Keywords:** cellular orientation, fiber alignment, neural tissue engineering

## Abstract

Tissues are commonly defined as groups of cells that have similar structure and uniformly perform a specialized function. A lesser-known fact is that the placement of these cells within these tissues plays an important role in executing its functions, especially for neuronal cells. Hence, the design of a functional neural scaffold has to mirror these cell organizations, which are brought about by the configuration of natural extracellular matrix (ECM) structural proteins. In this review, we will briefly discuss the various characteristics considered when making neural scaffolds. We will then focus on the cellular orientation and axonal alignment of neural cells within their ECM and elaborate on the mechanisms involved in this process. A better understanding of these mechanisms could shed more light onto the rationale of fabricating the scaffolds for this specific functionality. Finally, we will discuss the scaffolds used in neural tissue engineering (NTE) and the methods used to fabricate these well-defined constructs.

## 1. Introduction

Over the past 25 years, neurological disorders (ND) have been the leading case of disability and death worldwide [1]. According to the Global Burden of Disease Study (2015), ND is the leading cause of disability-adjusted life-years (DALY) in 2015 (229.1 to 274.7 million or 10.2% of global DALYs) and the second leading group of deaths (9.1 to 9.7 million or 16.8% of global deaths) [1]. ND such as Alzheimer’s accounts for the second highest number of deaths, whereas other motor neuron diseases still account for a fairly large number of deaths globally [1].

These neurological diseases involve the loss of neurons and synapses in various parts of the brain, spinal cord, and other parts of the all-encompassing peripheral nervous system. Despite the substantial decrease in mortality rates from stroke and communicable ND, its burden has increased in the past 25 years due to gradual increment of the aged population [1]. Hence, there is a need to prepare more efficient methods to provide adequate treatment for the ever-growing number of patients with ND.

The current treatment strategy for ND that involves neuronal loss, such as trauma to the spinal cord, is definitive surgical decompression and/or stabilization [2]. Autologous peripheral nerve graft has also been used as a treatment for Parkinson’s disease [2]. However, this procedure of stabilization requires the transference of nerve from another part of the nervous system, as seen in the excision of sural nerve containing Schwann cells and its delivery into the Parkinson’s disease affected substantia nigra [2]. The increment in morbidity to patients using surgical procedures drives the research for other avenues in ND treatment technologies.

As an alternative to nerve transplant, stem cell therapy provides a renewable source of auxiliary cells and tissues for a variety of ND [3]. Bone marrow cell transplantation has been used to treat spinal cord injury, and it is shown to be a viable option for patients with complete spinal cord injury [4]. However, the study shows only small improvements in the treatment of acute and sub-acute groups, but not in chronic groups [4]. This is may be due to the fact that the stem cells require a scaffold and vector to improve its functionality.

Biomimetic nanofibrous scaffolds has been developed for NTE in order to provide sustained growth factor/drug release or to support cell growth in situ [5,6]. The ability to fine tune the biochemical properties of the nanofibers enables researchers to produce biomaterials that could mimic the ECM of native tissues [7,8]. This potential coupled with a high surface area to volume ratio and superior biocompatibility provides a technique to reduce cell death or neuropathy due to nonphysiological local stress [8,9]. Many methods have been employed to fabricate these scaffolds according to the desired functionality and specifications.

Electrospinning is frequently used to fabricate scaffolds due to its ability to manipulate the developmental parameters such as porosity, surface area, fiber diameter, and its alignment therein [10]. It is considered as a standard technique for producing nanofibers in the field of NTE [11,12]. Another immerging technique to fabricate neural scaffolds is microfluidics [13,14]. This method requires no application of high temperature or voltage [15,16]. There are also other novel methods being developed to produce neural scaffolds, such as isoelectric focusing [17], wet spinning [18], and thermal drawing process [19]; with each technique improving the scaffolds attributes that could enhance and direct cellular growth.

Development of these fabrication techniques adheres to certain protocols when it comes to scaffold production for tissue engineering purposes. Regardless of tissue types, a sustainable scaffold needs to be biocompatible and biodegradable [20]. Mechanical properties of the scaffolds are also an important consideration because the various culturable cells requires ECM of different stiffness for efficient growth [20]. Scaffold architecture such as the level of porosity and its interconnectivity has to be taken into account for 3D cell culture [20]. Finally, the methods used to fabricate these scaffolds has to be cost effective and up-scalable [20].

The fabrication of neural scaffolds applies these criterion guidelines in a more cell specific manner. Neural cells respond to a distinct topological cue, where the neuronal outgrowth needs to be guided and the connection between neurons has to be established for efficient growth [21]. Bearing this in mind, the scaffolds should mimic the native tissue ECM’s topological, mechanical, biochemical, and electrical cues to promote better contact guidance, adhesion, and proliferation of neuronal cells [21]. The radical scavenging ability should also be incorporated in to the scaffolds to minimize secondary progression of injury [21].

These scaffolds that are shaped and solidified in vitro requires surgical insertion into the affected site. A more immediate method of therapy would include on-site treatment to reduce scarring or accumulation of inhibitory proteins. Hence, in recent years, researchers look to injectable hydrogels as a viable option for minimally invasive treatment of various injuries [22,23,24]. These hydrogels could be administered immediately after injury, and since it does not require surgery to apply the scaffold, the morbidity from trauma would be greatly reduced [25].

However, injectable hydrogels do not have defined microarchitecture that is found in in-vitro patterned scaffolds [26,27,28]. This topology on scaffolds plays an important role in orientating cellular growth and building appropriate micro-structures that could provide sustained tissue development [26,27,28]. Current advancements in this new class of hydrogels employs nanoparticles and nanotubes to direct polymer fibers therein and in effect, direct the alignment of cells [26,27,28].

In this review, the examination of currently used hydrogels for NTE is presented. The recent methods of fabricating scaffolds with aligned microarchitecture is also outlined; where it ranges from the most commonly used techniques hitherto such as electrospinning to more novel ones that are currently being developed. Overall, the processes that leads to cellular alignment within these scaffolds or during the fabrication procedure are reviewed.

## 2. The Common Characteristics of Hydrogels Used for NTE

**Figure 1:**
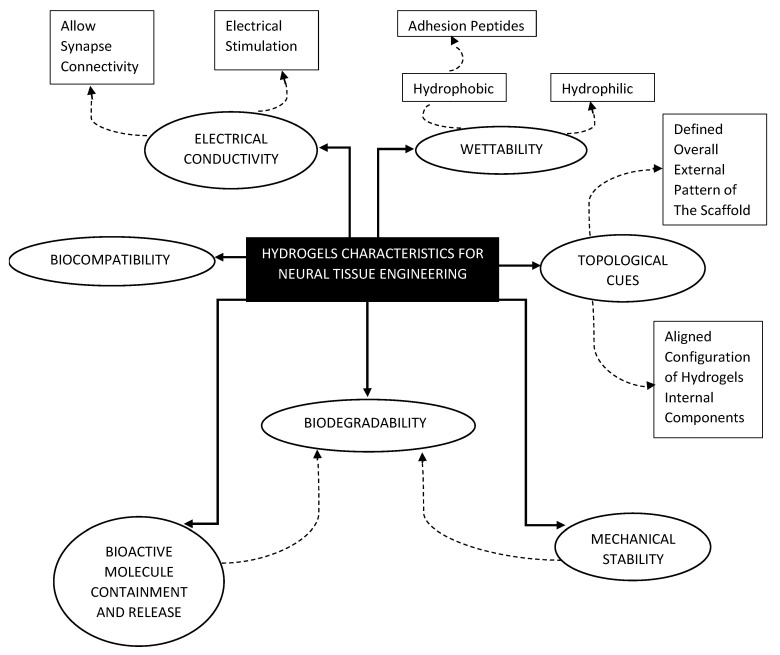
Desired characteristics of hydrogels chosen for scaffold fabrication.

NTE specifically focuses on the strategies to promote nerve regeneration and the proper restoration of an injured central nervous system (CNS) or peripheral nervous system (PNS). The behavior of nerve cells differs from other cell types; even between the CNS and the PNS [29]. It has been shown that nerve cells are capable of bridging gaps under 6 mm [30]. Any injury that is larger would necessitate the use of scaffolds. Consequently, materials chosen to fabricate these scaffolds should possess attributes that caters to enhancing the regeneration capabilities of nerve cells. Figure 1 shows a chart that summarizes the common characteristics of neural hydrogels.

One of the most important criteria for choosing hydrogels to make scaffolds is biocompatibility and biodegradability [30]. Chronic inflammation could become a serious issue during the recovery period and it could worsen the patient condition [30]. To eliminate this problem, a biocompatible scaffold is used to limit the patient’s own resistance to this foreign element and enable the healing process to take place more efficiently [30]. This hydrogel should also be biodegradable to enable patients own cells and tissues to replace the loss experienced during injury [30].

For cells to adhere properly to the scaffold, the hydrogels used need to possess a certain level of wettability [31,32]. Synthetic materials that are biodegradable such as poly (lactic-co-glycolic acid) (PLGA) and polycaprolactone (PCL) are hydrophobic and this limits its suitability as a viable hydrogel for scaffold production [31]. However, surface modification could be done, through the incorporation of specific adhesion peptide sequences such as Arginylglycylaspartic acid (RGD) or coating the surface of the scaffold with ECM proteins such as laminin and fibronectin [31]. This improves the scaffolds hydrophilicity and in turn, cellular adhesion.

Mechanical stability is an important aspect of scaffold design [33]. Components of the ECM helps support and give structure to the tissues, and hydrogels that are chosen for scaffold fabrications should be able to mimic this function. When it comes to the structure stability of scaffolds, biodegradability has to be taken into account as it effects how long the mechanical integrity of the construct could be maintained before the recovered tissue could take over [33]. To enable this, hydrogels are preferably chosen based on its surface erosion potential compared to its bulk erosion potential, since it allows fabricated scaffolds to retain their structure a little longer after treatment [33]. Researchers have also studied composite polymers to improve the mechanical stability of softer, more biocompatible hydrogels [34].

During the tissue repair process, it is common for the cells to release growth factors as signaling molecules [35]. If it is a serious injury, the amount of cell loss and accumulation of inhibitory proteins may prohibit signaling and hence, the healing process is affected [36]. In such cases, it is crucial that the transplanted scaffold retain the concentration of these growth factors at the injury site [37]. With the discovery of neural growth factor (NGF), nerve cell regeneration could be enhanced, to avoid the formation of glial scar [38,39]. Polyphosphoesters have also been used to protect NGF from the acidic microenvironment at the site of trauma [36]. These advancements in hydrogel’s growth factor delivery allows the healing process to be much more efficient and effective.

Another important hydrogel characteristic used for neuronal cell culture is electrical conductivity [40]. Neural communication within the human body involves action potentials that are generated at the synapses [40]. Hence, to promote neuron signaling and outgrowth, the implanted scaffolds must conduct or even improve this communication between cells [41,42]. Taking this concept even further, researchers applied electrical stimulation in order to enhance nerve regeneration on polypyrole scaffolds [42]. In vivo studies using conductive composite scaffolds made from polypyrole, poly-DL-lactic acid and polycaprolactone to bridge an 8 mm gap in rat sciatic nerves shows the enhancement of nerve cell proliferation and axonal regeneration through the application of electrical cues [43].

Topological cues from the ECM plays an important role in cellular adhesion, proliferation, and differentiation [44]. This primarily involves the subtle arrangements of ECM structural proteins in a specific manner that enable the manipulation of cellular growth and fate [45]. Therefore, the hydrogels chosen for NTE should be based on its ability to guide neurite growth and axonal expansion [46]. The focus on scaffold microarchitecture construction could be seen from a two-dimensional perspective with the fabrication of defined nanostructures such as grooves, and ridges on the surface or from a 3D perspective with fabrication process that could directly alter the alignment of internal nanofibers [37,44,46].

Due to its resemblance to native tissues ECM, nanofibers have become a promising substrate to construct scaffolds [46]. Polymers specifically chosen for NTE usually could produce nanofibers of exceptional mechanical strength and high surface area to volume ratio [47]. Thus, various types of scaffolds are fabricated from hydrogels ranging from natural and synthetic, to composite materials, or even the incorporation of nanoparticles to produce nanofibers that could mimic in vivo cellular structure.

## 3. Process of Axonal Alignment in Neural Cells

Understanding the natural cell’s position within its tissue structure is crucial in the development of functional scaffolds. This cellular positioning not only plays an important role in altering the topological cues, but also controls its interaction with the ECM, and the biochemical signaling that ensues [47,48]. This is especially true for the neural cell types; whose primary role involves sending signals to far areas within our body. Hence, its directionality should be given top priority when determining the criterion of a neural scaffold’s development for proper differentiation and maintenance of cellular morphology and functionality. Why is this cellular alignment so important though? To understand this, we have to take a look at the cells mechano-sensing and cytoskeletal dynamics that changes as it come in contact with its ECM microenvironment for adhesion, support, and alignment [49]. This also applies to the cell’s adaptation to the mechanical stiffness of neural scaffolds and how it mimics natural healing [49].

Axonal alignment is the result of subsequent rearrangement of microtubules and F-actin proteins within the axon’s growth cone following the cells adherence to a substrate [50]. This external mechanical force effects every cell that attaches to a substrate [50]. It is also worth pointing out the balancing internal cellular force acting on the cell’s membrane which is brought about by the contracting filopodia as well as the cytoplasmic and cytoskeleton pushing forces [51]. This balancing act of external and internal forces allows cells to attach itself onto substrates and form neuronal synapses, controlling the length of neurites and its orientation [49]. The rise in axoplasm viscoelasticity is caused by the polymerization of microtubules and actin, which increases the length of axons [52]. Research has shown the improvement in growth and functionality of neural cells that were cultured in scaffolds that mimics the stiffness of local neural tissue matrix, and the reason for this was primarily attributed to the mechano-sensitive ion channel called piezo-1 [53]. This piezo-1 channel has a level-like mechano-gating mechanism that, when moved by an external traction force such as cellular adhesion, would allow the influx of calcium into the cells which then regulates the growth and differentiation capabilities of neural cells [54].

**Figure 2:**
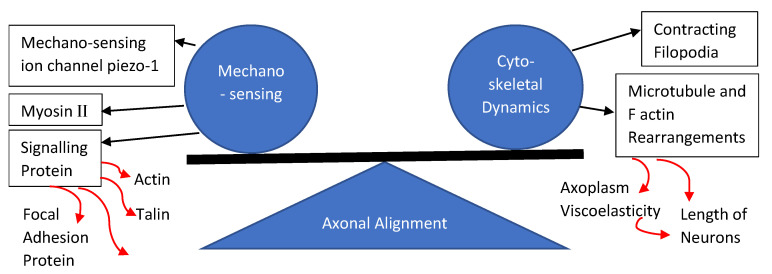
The process of axonal alignment in neural cells.

The other constituent of axonal extensions such as actin, microtubule and microfilament proteins, as well as their arrangement and reaction to an external mechanical stress should also be considered to really understand the cells capability for alignment [55]. Microtubules, which are much more rigid than actin, plays a part in influencing the stiffness of neurites by crosslinking with actin [56]. The microtubule-actin complex is attached to the cellular membrane via adaptors and signaling protein that could alter the actin polymerization process and guide the growth of axons [57]. This cone of neurite expansion also contains myosin II which regulates the severing of polymerized actin [58]. Signaling proteins such as talin, paxillin, focal adhesion kinases, and A-actin play an important role in the substrate-cell actin dynamics [59]. The mechanical stress acting on the cytoskeletal dynamic eventually leads to neuronal mechano-sensing [60]. External mechanical forces are converted into biochemical signals via mechanically activated ion channels such as transient receptor potential ion channel, acid-sensing ion channels, and piezo ion channels [60]. Figure 2 shows the summary of the axonal alignment process in neuronal cells.

## 4. Methods of Scaffold Fabrication That Promotes Axonal Alignment

Imitating the ECM structure of cells has been an integral part in the design of neural scaffolds. Neural cells require aligned nanostructure to grow in, due to its function in signal transduction. Such scaffolds with precisely regulated topology orientate the growth of neural cells and its dendrites, which in turn, guides the pathway of the signal. Therefore, technologies that produces neural scaffolds with aligned nanostructures is crucial in the proper development of neural cells that are cultured therein. This internal nanostructure of scaffolds could be mirrored by the use of self-assembling fibrous materials or materials that are made fibrous using certain fabrication techniques. The most utilized method in recent times to produce scaffolds is electrospinning. Microfluidics and bioprinting have also been widely used within this field in these past couple of years. However, there are also new methods, such as magnetic orientation and isoelectric focusing, that has been developed to accommodate the different strategies in neural scaffold fabrication. Table 1 shows a summary of modified electrospinning methods used to produce NTE Scaffolds.

### 4.1. Electrospinning Technique

**Figure 3:**
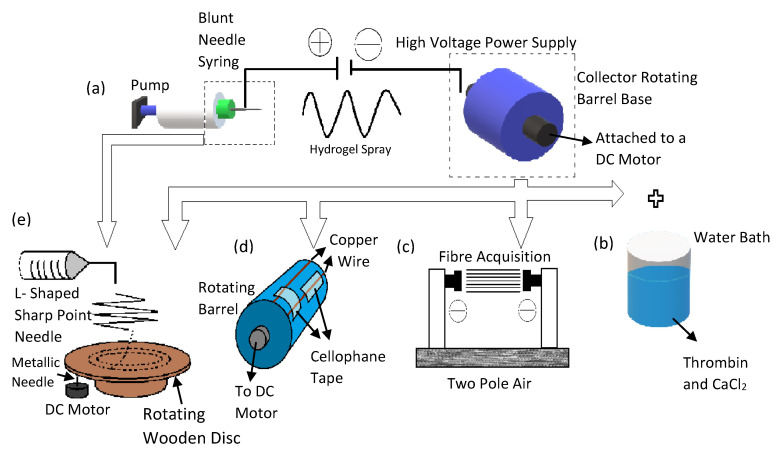
This diagram illustrates the electrospinning process and its numerous modifications seen in neural scaffold fabrication. (**a**) The basic electrospinning setup. (**b**) Electrospinning combined with the wet spinning setup. (**c**) Two pole air gap spinning. (**d**) Modifications made to the rotating drum to produce scaffolds of dual morphology. (**e**) New novel electrospinning design that uses a rotating wooden disc to collect fibers.

**Figure 4:**
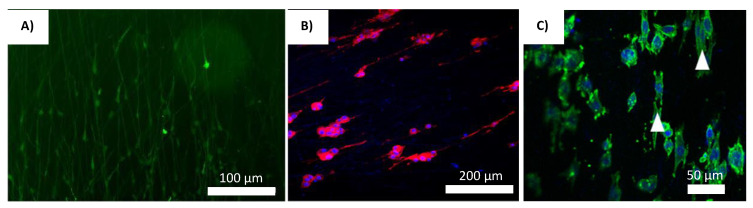
Cells cultured on different electro-spun scaffolds. (**A**) Primarily the GFAP+ cells migrated along the fibers in straight lines [64]. Reprinted with permission copyright © 2017 by authors and Scientific Research Publishing Inc. (**B**) PC12 cells on LLA-GO nanofibrous scaffolds coated with 1.0 mg/mL GO solution [65]. Reprinted with permission copyright © 2016 Acta Materialia Inc. Elsevier Ltd. (**C**) DPSCs on PLO-coated aligned PCU fibrous scaffolds [66]. Reprinted with permission copyright © 2017 Elsevier B.V.

Ever since research groups such as Reneker and Rutledge shown the world that it is possible to electro-spin organic polymers, there has been a rising trend in the application of electro spun materials in the field of biomedical engineering [61]. Hence, considerable research has been done to improve the controls on the minutiae of the fiber producing technology, such as defining the structure of the Taylor cone (Point of eruption at the needles nozzle) and the various instabilities of fiber generation rates in relation to the voltage applied [62,63]. The basic design of the electrospinning device has been vastly improved since its inception. This improved design has been used as the gold standard in many NTE studies. It mainly comprises of a ventilated hood, a spinneret (which is usually a hypodermic syringe), with a blunt syringe needle attached, a high voltage supply, and a ground collector, as seen in Figure 3a [64,65,66]. Researchers Ulrica et al. used this set up to investigate the cellular migration and phenotypic differentiation behavior of human neuron progenitor cells (HNPC) in electro spun PLLA scaffolds [64]. In order to produce fibers of various morphologies, the high voltage power supply was attached to the blunt needle at two different locations [64]. Analysis of the nuclei orientation shows it was within 20° of the fiber angles [64]. The fabricated fibrous PLLA scaffolds greatly influences the migration pattern, enhances the potential for differentiation, and nuclei polarization of the HNPCs [64]. The cells orientation in the scaffold is shown in Figure 4A.

Zhang et al. utilized a similar electrospinning setup to produce aligned PLLA/graphene oxide (GO) scaffold that was used as a structural foundation to direct cells orientation, and further support cellular growth [65]. The configuration consists of the standard setup with the addition of a rotating drum used as a ground collector [65]. These electrospun scaffolds were then aminolyzed to prepare it for the addition of GO, which was previously prepared using Hummer’s method [65]. The coating of GO on the aligned topology of the nano fibrous PLLA scaffold improved the surface roughness, hydrophilicity, and enhances cellular attachment to the scaffold [65]. The regulated directionality of the PLLA fibers improved the growth of Schwann cells (SC) as well as directed its orientation in situ [65]. This enhancements in differentiation capabilities are also exhibited by PC12 cells, leading to its neurite like growth on the scaffolds [65]. These PC 12 cell also shows highly aligned neurites on the PLLA-GO scaffolds [65]. The cell orientation in the scaffold is shown in Figure 4B.

Another novel modification was made to the electrospinning method by researchers Lee et al. to produce highly aligned fibrous neural scaffolds using the thermoplastic, polycarbonate urethane (PCU) [66]. An aluminum foil-coated rotating mandrel was used to collect the fibers jetting out of a blunt needle [66]. The mandrel’s rotating speed was used to alter the alignment setting of the scaffolds [66]. The scaffolds were also coated with bioactive compound, Poly-l-Ornithine (PLO) to improve cellular attachment and directionality [66]. The results obtained showed that the highest speed of mandrel rotation (1800 RPM) produced the most aligned PCU fibers [66]. With the addition of other bioactive agent PLO, dental pulp stem cells (DPSC) presented greater attachment and proliferation along the fiber alignment path [66]. The cell orientation in the scaffold is shown in Figure 4C. This illuminates the synergy between the directed fibers and improved attachment of cells via bioactive compounds.

An innovative two pole air gap electrospinning technique was used by Nguyen et al. to produce highly fibrous scaffolds using poly(ε-caprolactone-co-ethyl ethylene phosphate) (PCLEEP) (shown in Figure 3c) [67]. Basically, the PCLEEP polymer solution was charged and jetted out of the blunt needle tip in to an air gap area where it will be deposited between two stationary collector poles [67]. These fibers were used as a foundation to fabricate the final scaffold in which, they were embedded at the core of collagen cylinders [67]. Several drug or genes were also loaded into these scaffolds such as neurotrophin-3 (NT-3), and miRNA to determine the efficacy of this scaffold in controlling the release of these neurite enhancing agents [67]. When the scaffolds were implanted into the incised portion of rats C5 spinal cord, there was effective alignment of neurite outgrowth as well as its remylenation in situ [67]. This is attributed to the highly aligned topographical cues provided by the electro spun scaffolds during the healing process [67]. There were also greater cellular attachment potential and drug delivery control, which was provided by the collagen cylinder shells of the scaffolds [67].

The neural scaffolds developed by Kim et al. has both random and aligned fibers but this unique combination was capable of directing the growth of PC12 and S24 cells [12]. The aligned fibers spun in the inner surface of the scaffolds orientates neuronal cells and the randomly aligned fibers on the central part of this nerve graft strengthens the weaker aligned structure [12]. The modified design of this electrospinning set up involves a conductive rotating collector with cellophane tape attached copper wires (shown in Figure 3d) [12]. The cellophane tapes were adhered horizontally and vertically onto the copper wires of the rotating platform which then produces fibers of two different alignment simultaneously [12]. The polymer that was used in this research was PLGA and Poly Urethane (PU) [12]. The PC12 cells culture in these scaffolds showed greater proliferation on the aligned inner surface compared to the outer, randomly aligned fibers [12]. It was also noted that due to the higher porosity of the inner aligned layer, there were greater cell adhesion [12]. There was also greater alignment in this part of the scaffold [12]. However, these results were not observed in the S24 cell culture study, due to the cells smaller size and hence its inability to inhabit a larger substratum [12].

Another unusual but highly effective method of electrospinning sees the use of a wooden disc as a collector [68]. The control of electro spun scaffolds properties such as degree of alignment and fiber thickness done using this novel wooden disk electrospinning set-up shown by researchers Vimal et al. involves an L shaped sharp point needle to direct the polymer solution downward onto a rotating wooden disc (shown in Figure 3e) [68]. In order to produce scaffolds of various fiber alignment, several parameters could be tweaked, such as the radial distance of fiber attachment on the spinning wooden disc, voltage applied, rotational speed, and distance between the needle and disc [68]. The polymer solution used for this study was polystyrene mixed with tetrahydrofuran (THF) and dimethylformamide (DMF) [68]. Upon optimizing the electrospinning parameters to produce scaffolds of highly aligned, moderately aligned and non-aligned fibers, human astrocytoma cells (U373) were used to evaluate its efficacy in cellular culture [68]. The results obtained shows that scaffolds of both moderately and highly aligned fibers have greater cellular orientation; and the degree of alignment is improved as the fibers directionality gets more regulated [68]. It was also shown that the highly aligned fibers provided better contact guidance compared to the other scaffold types as evidenced by the prominent number of cells present therein [68].

Synthetic materials have been used more often in the electrospinning process due to its mechanical strength and tailorable properties. However, natural materials could be utilized if the electrospinning setup is altered to accommodate these fragile hydrogels. This application is shown in the research done by Yao et al. and Du et al. [69,70]. Here fibrin was used due to its role in the natural healing process of peripheral nerve injury [69,70]. Fibrin is formed from the self-assembly of fibrinogen in the presence of thrombin [70]. Therefore, the electrospinning set up was altered to incorporate the wet spinning process by including a rotating collector water bath containing calcium chloride (CaCl2) and thrombin (shown in Figure 3b) [69,70]. This fibrin electrospinning set up was based on a research done by Yao et al., where the highly aligned electro spun fibrin was used in conjunction with the effects of its matrix low elasticity to enhance the neuronal differentiation capabilities of human mesenchymal stem cell (hMSC) and promote its outgrowth [69]. Results obtained from the in vitro test showed the formation of neurons from the differentiation of hMSCs, where they aligned and proliferated along the fibers length, without the use of neurotrophic agents [69]. When the scaffolds were implanted in rats with T9 dorsal hemisection spinal cord lesion, there was significant axonal outgrowth from the cells cultured therein, which shows enhancement in the amelioration effects of these scaffolds during the healing process [69]. Researchers Du et al. used this electrospinning setup to produce fibrin cables that guides the growth and migration of Schwann cells and aid in directed axonal regrowth [70]. The fibrinogen itself was mixed with poly (ethylene oxide) (PEO), but was washed out later with PBS after fibrin formation [70]. Schwann cells grown on these scaffolds showed remarkable orientation and attachment [70]. When used for bridging the long sciatic nerve gaps in rats for the in vivo evaluation, the electro spun fibrin scaffolds were combined with chitosan tubes [70]. By comparing this scaffold with plain chitosan tubes or randomly aligned fibrin scaffolds, it was disinterred that the axonal regrowth was much faster in groups with the combined aligned electro spun fibrin scaffold/chitosan tube. It also enhanced the morphogenesis of the cells as well as its motility function in situ [70].

The use of the electrospinning technique has brought about various improvements to the design of neural scaffolds as discussed above. However, there are also a number of limitations that needs to be addressed. The most prevalent drawback is the requirement of surgery for scaffold application. The scaffolds fabricated with this technique are usually solid and firm in structure; hence, a greater degree of fiber alignment in these constructs could be maintained [64,65,66,67,68,69,70,71]. However, to utilize the scaffolds, an incision would need to be made at the site of injury to patch these firm scaffolds thereat, which not only increases the rate of morbidity but also makes it difficult to apply the scaffolds when immediate response is required for treatment, as seen in blunt spinal cord trauma.

Mechanical strength of the fibers produced is also an important feature for scaffolds fabricated using the electrospinning technique and as these strings are spun in layers, where the lower structure acts as a foundation for subsequent fiber coatings. Hence, many researchers opt to use synthetic materials exclusively, which limits the types of biomaterials applied with this fabrication method [64,65,66,67,68]. If natural materials are used, it would usually require a sheath to hold its structure, making it more complicated and costly to develop. However, some researchers such as Kim et al., improved the design of these scaffolds by adding an aligned inner layer and nonaligned outer layer [12]. Other innovations have also been made such as the incorporation of a rotating water bath from the wet spinning technique, which enable the use of natural materials such as fibrin for micro fibrous scaffold fabrication [69,70]. This goes to show that with further modifications, the range of biomaterials useable with this technique could be expanded.

It is also important to note that inclusion of cells within the hydrogels during the scaffold fabrication process could not be done using the electrospinning technique. This is due to the harsh environment involving high voltage and the presence of solvents during the spinning process that might not make it suitable for cell incorporation. The inclusion of cells may enhance its orientation even further since acclimatization of cells to the material could occur before scaffold fabrication. Adding cells in prior to scaffold fabrication could also evenly distribute the cells throughout the structure. However, with the electrospinning technique, cells could only be added after the scaffold is already fabricated. This could be an area in which researchers could further expand upon the functionality of the electrospinning technique to include cells during the fabrication process.

### 4.2. Microfluidic Technique

Microfluidics technology is another avenue researchers’ have explored in the pursuit of fabricating neural scaffold capable of orientating cellular and axonal growth. Compared to electrospinning, microfluidics chip design is usually custom, and its setup vary between most of the research groups utilizing it. However, the key component in building a microfluidics device involves the control of polymer flow as well as its crosslinking process through the corresponding channels within enclosed chambers. Some of these devices could even act as bioreactors to sustain the growth of cells and manipulate its orientation and differentiation. Table 2 shows the summary of modified microfluidic fabrication method to produce scaffolds for NTE.

**Figure 5:**
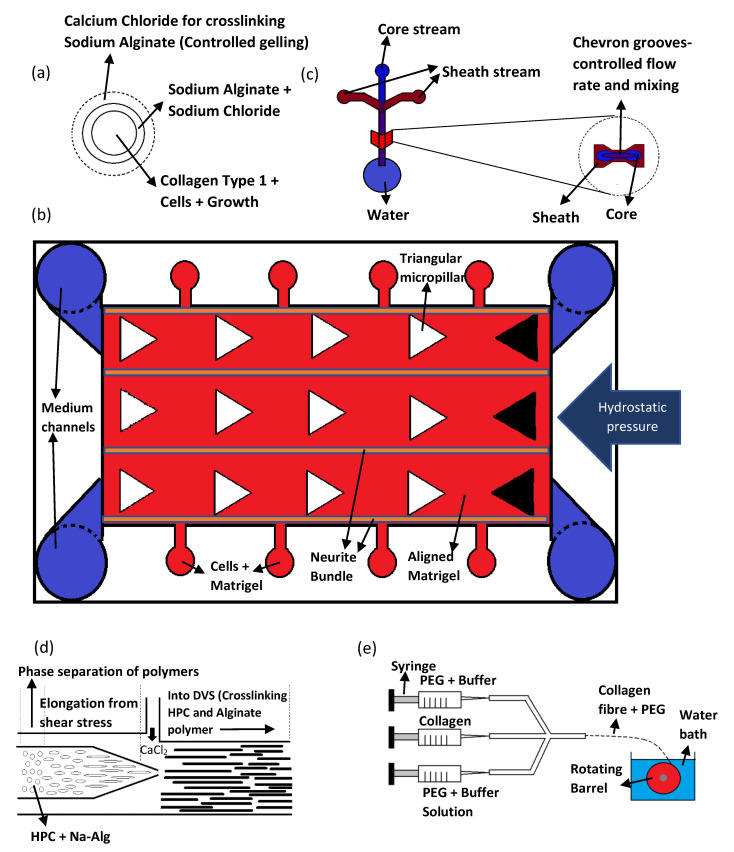
Schematics of various microfluidics devices. (**a**) Cross-section of the microfluidic channel. (**b**) A top view of the microfluidic device. (**c**) The cross-section of the chevron grooves. (**d**) A side way cross-section of the microfluidic channel. (**e**) The microfluidic setup coupled with a wet pinning method.

Researchers Kato-Negishi et al. utilized a microfluidic device with double coaxial laminar flow to fabricate rod shaped neural units [71]. This flow comprises of three layers, namely, the core stream, shell stream, and sheath stream (shown in Figure 5a) [71]. The core stream contains cortical cells or hippocampal cells, along with collagen type 1 and Dulbecco’s modified eagles’ medium (DMEM) [71]. The sheath stream consists of sodium alginate in sodium chloride which maintains the mechanical integrity of the underlying collagen scaffold [71]. Finally, the shell stream contains calcium chloride which is required for crosslinking the sodium alginate after the shape of the scaffold is established [71]. The flow force from the microfluidic device was used to align the collagen fibers and cells in the core stream, while the sodium alginate holds the microfiber shape of the resulting scaffold [71]. The sodium alginate also acts as an insulator to fence the proliferation of cells within the core strand [71].

Microfluidic devices could also be designed to simulate 3D neural circuits. In another study by Bang et al., a microfluidic device was used to simulate 3D neural circuits using rat cortical neurons [72]. The design of this device is based on arrays connected by channels containing micropillars as shown in Figure 5b [72]. These arrays are loaded with cells, growth medium or hydrogels, and is allowed to flow into the five microchannels in between the arrays in a controlled manner [72]. The micropillars are made triangular to prevent air bubble formation during scaffold fabrication [72]. The channels were filled with Matrigel and hydrostatic pressure was applied from the two arrays on one side of the device [72]. This hydrostatic pressure aligns the ECM components of the Matrigel as well as the cells that are cultured therein [72]. Furthermore, the architecture of the micropillars and channels enables controlled crosslinking of Matrigel which configures the formation of the hydrogel layers by interchanging between zones of compact and sparse density [72]. Since neurite proliferate towards the sparse area of the Matrigel, this configuration directs the growth of cells in the direction of medium flow [72].

**Figure 6:**
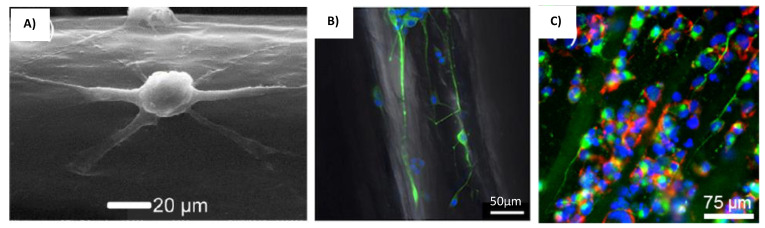
Cells cultured on different microfluidic scaffolds. (**A**) SEM images of the AHPCs cultured on the PCL microfibers [73]. Reprinted with permission copyright © 2016 American Chemical Society. (**B**) PC-12 cells cultured on the bundle gel fibers [74]. Reprinted with permission copyright © American Chemical Society. (**C**) Neuronal NG108-15 cells on microfluidics-produced collagen microfibers [75]. Reprinted with permission copyright © 2016 American Chemical Society.

The microfluidic technique could also control the fiber alignment by changing the flow rates of the core and sheath fluids. This was shown in a study by Sharifi et al., where a microfluidic device with triple chevron grooved channels was used to fabricate micro fibrous polycaprolactone scaffolds capable of guiding the growth of adult hippocampal progenitor cell (AHPC) [73]. The microfluidic device itself was made with PDMS using SU8 photoresist-patterned silicon wafer mold [73]. Three microchambers for the core and sheath fluids were linked together by microchannels that has four chevron grooves along a part of its length (shown in Figure 5c) [73]. This differentiate the flow rate of core and sheath fluids, which were PCL and PEG, respectively [73]. This mechanism maintains the fibers alignment after fluid injection [73]. It was also reported that the diameters of the fibers could be altered by manipulating the sheath and core flow rate [73]. However, the PCL fibers have much weaker mechanical properties at higher flow rates [73]. Therefore, in order to produce stronger fibers, its diameter was limited by the flow rate [73]. The AHPC cell did not deviated from the axial direction within the fibers fabricated with any of the sheath-core flow rate ratio [73]. The SEM images of the AHPCs cultured on the PCL microfibers is shown in Figure 6A. However, using a sheath-core ratio of 300:2 and 200:4 produces a higher percentage of cells deviating from this directed path, and is attributed to the bridging of cells with the microfiber bundles [73].

Materials that have different phases and crosslinking properties have also been used with the microfluidic method to direct the flow of the hydrogels and produce highly aligned multi-fibrous scaffolds. Tachizawa et al. uses a co-flow microfluidic device prepare scaffolds made of hydroxypropyl cellulose (HPC) and sodium alginate (Na-Alg) [74]. This device has an inner and outer channel where the mixing rate of polymer and crosslinker may be altered accordingly, as shown in the cross-section diagram in Figure 5d [74]. The crosslinker from the outer channel crosslinks the Na-Alg to give some mechanical stability to the resultant fibers [74]. The scaffolds are then dipped in divinyl sulfone (DVS) to crosslink the HPC and alginate polymer [74]. The microfibers are then immersed in citric acid to remove uncross-linked alginate [74]. The internal aligned multi-fibril architecture was created by the shear stress within the laminar flow, and the phase separating properties of the polymers (HPC and Na-Alg) [74]. Basically, the flow force elongates the phase separated polymer mix to create bundled gels [74]. The polymer blend ratio also effectively controls the alignment and stiffness of the microfibers produced [74]. A higher Na-Alg concentration allows the formation of single bundled micro fibers which are smaller and stiffer [74]. This guides neuron cells along the fibrils and permits the proliferation of its axon along the length of the fibers [74]. The cells orientation image within the scaffold is shown in Figure 6B. Additionally, the human IPSC-derived dopaminergic neuron cells grown within these scaffolds successfully differentiated into mature neuron cells [74].

Researchers Haynl et al. has also utilized a microfluidic chip to fabricate collagen fibers with good mechanical strength for neuronal cell line NG108-15 culture [75]. The microfluidic chip was fabricated with PDMS using soft lithography technique [75]. It has a similar core-sheath design which includes a core inlet channel for the collagen solution and another two sheath channels containing buffer solution and PEG [75]. The fibers produced by the microfluidic chip was collected by a rotating spool in a water bath (shown in Figure 5e) [75]. To avoid clogging, a tiered design was used to construct the flow tubes [75]. PEG was utilized as a hygroscopic agent, which improves the rate at which the protein precipitates and form fibers [75]. The PEG was washed-off along with the buffer solution in the water bath before the fibers are spun up on to the rotating spool [75]. The fibers that were produced have high mechanical strength and is capable of directing the migration and growth of neuronal NG108-15 cells as well as support its axonal development along the microfiber’s orientation [75]. The cells orientation image within the scaffold is shown in Figure 6C.

Microfluidics is a promising new technique to produce highly aligned micro fibrous scaffolds using different polymers, be it natural, synthetic, or a blend of both. The fabrication concept revolves around the control of the polymers gelling point which could be achieve using microfluidics in many different ways, such as altering the flow rates of cross linker and polymers, or by mixing polymers with different gelling properties to align internal microarchitecture and maintain structural integrity. However, these microfluidics chips need to be designed and fabricated first before the fibrous scaffolds could be produced, which may drive up manufacturing cost and time. The procedures reviewed here mainly utilizes PDMS microfluidic chips made from silicon wafer molds which could reduce the manufacturing cost. It is also important to note that the miniscule design of the microfluidics channels within microfluidics chips and the application of hydrostatic pressure makes clogging a serious problem, especially when multicomponent polymers such as Matrigel is used. Therefore, special care needs to be given when designing the microfluidic channels as well as the pressure applied therein, especially when fabricating scaffolds with finer fibers.

### 4.3. 3D Bioprinting

The advent of 3D bioprinting technology that uses rapid freeform prototyping, enables quick manufacturing of biological 3D structures compared to other conventional fabrication methods [76]. The methodology of printing 3D computer schematics using specifically made bio-ink could produce scaffolds that have uniformly distributed cells as well as the flexibility in positioning these cells anywhere along the construct [77,78]. Table 3 shows a summary of bioprinting-based fabrication methods to produce scaffolds for NTE.

**Figure 7:**
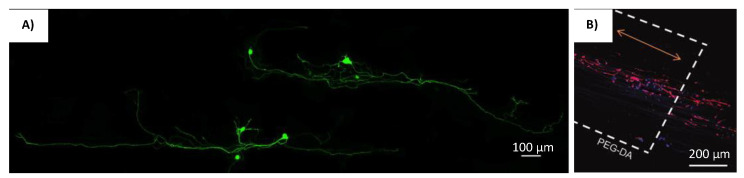
Cells cultured on different bio-printed scaffolds. (**A**) DRG neurites aligned along 3D printed fibrin-factor XIII-HA strands [79]. Reprinted with permission copyright © 2016 Elsevier B.V. (**B**) Neurite growth of primary cortical neurons on scaffolds with PCL/gelatin fibers [81]. Reprinted with permission copyright © Mary Ann Liebert, Inc.

In recent years, 3D printing technology has been utilized to fabricate scaffolds for NTE applications; more specifically scaffolds with intricate microarchitectural designs to guide the growth of axons and neuronal cells. Researchers England et al. made use of an extrusion-based 3D bioprinting procedure which utilizes a viscous bio ink made up of hyaluronic acid (HA) and poly-vinyl alcohol (PVA) [79]. To further increase the rate at which the bio-ink solidifies, clotting factor XIII was used [79]. Therefore, the use of fibrin was made possible despite its low viscosity [79]. The inclusion of this fibrous protein provides the guided pathways that directs axonal alignment and cellular growth [79]. The Schwann cell encapsulated within these scaffold shows dorsal root ganglion neurite growth along the longitudinal alignment of the fibrin fibers, which provides haptotactic cues for cellular orientation [79]. The cells orientation image within the scaffold is shown in Figure 7A. However, these results could not be replicated with other carbohydrate-based hydrogels, which indicates the importance of fibrin self-assembly within these constructs [79].

Maintaining the scaffolds 3D structure during and after printing could also be done using photolithography. This procedure was applied by Li et al. to produce scaffolds made up of polyethylene glycol diacrylate (PEGDA), methacrylated gelatine (Me-Gel), and methacylated hyaluronic acid (Me-HA) with detailed microchannels using digital light processing (DLP) based 3D printing [80]. These microchannels were also incorporated with a gradient of stromal derived factor-1 (SDF-1) to promote neural stem cell (NSC) migration into the microchannels [80]. It was shown in this research that the SDF-1 induces chemotaxis within NSCs to adhere and grow around the microchannels [80]. These microchannels with well-defined orientation would then align the axonal growth of the NSCs along its length [80].

Researchers Lee et al. used highly aligned electro spun scaffolds as a foundation for bioprinting neural scaffolds [81]. PCL/Gelatine was used to create the highly aligned fibrous scaffold foundation [81]. A standard electrospinning setup was used, with the PCL/Gelatine solution at 50:50 (*w*/*w*) was jetted out of a 21G blunt needle on to a rotating mandrel [81]. This was then coated with a mixture PEG (40%) and PEG-DA (60%) using 3D printing [81]. It was shown that the 3D-coated fibers enhanced NSC’s cellular proliferation and differentiation [81]. The PEG-DA on the other hand has great porosity which enhances cellular attachment; hence its use as the coating material for the electro spun PCL/Gelatine scaffolds [81]. The alignment of NSC’s was most efficient in scaffolds with the PCL/Gelatine foundation [81]. The cells orientation image within the scaffold is shown in Figure 7B. Therefore, the combination of fabrication techniques allows for the mixture of different materials, that enables the production of highly aligned fibrous scaffolds with 3D printed porous external structure [81].

3D coaxial printing is another method used by researcher that combines a microfluidic approach with the exactness of computer aided scaffold fabrication [82]. In this particular study by Sanz, photolithography was also used to immediately solidify the scaffolds post fabrication [82]. Collagen methacrylate (ColMA) and gelatine methacrylate (GelMA) were used as bio-ink to produce a model of neuromuscular junction (NJM). However, instead of mixing these hydrogels together, they were extruded from different nozzles to produce a construct made up of two separate materials that specializes in the culture of different cell types [82]. The methacrylation of collagen for immediate UV crosslinking after scaffolds fabrication improves its mechanical integrity. The shell component of the scaffold was made up of fish derived collagen and NSC-34 (motor neuron-like cells), whereas the core bio-ink was made up of GelMA and primary myoblast cells [82]. It was reported here that the NSC-34 cells were producing neurites that grows from the shell towards the core layer, which follows the orientation of sacromerin-actinin expressing myofibers [82]. This extension towards the area of skeletal myoblast localization forms the motor neuron-skeletal muscle contacts and NJMs [82].

In summary, the use of bioprinting for NTE applications could yield scaffolds of complex design and functionality. There are various strategies for aligning axons of neural cells cultured within these scaffolds, such as the use of self-assembling fibrous biological materials, constructing microchannels, or even co-culture of cells within different hydrogel’s microenvironment. The design of these scaffolds could be planned and its fabrication could be executed with great detail. However, this level of spatial control comes at a hefty monetary cost. The fabrication of these complex scaffolds necessitates the use of expensive bioprinting machines. Even if more affluent researches could procure a bioprinting device, there is still the question of finding an appropriate bio-ink that could be used in this procedure. This bio-ink should have the appropriate viscosity to retain its shape during the fabrication process, or at least long enough for the post fabrication to solidify the scaffolds. Furthermore, to produce aligned microstructures for axonal guidance, often times other fabrication methods such as electrospinning and microfluidics have to be used. The post fabrication process may also involve harmful chemicals or procedure that could harm cell growth.

### 4.4. Magnetic Orientation

Research on injectable hydrogels have increased in recent years due to its low morbidity and minimally invasive application. The ability to form complex and regulated patterns inside a living organism has driven researchers to develop techniques that enable them to manipulate the precise gelling point and formation of microstructures with these hydrogels remotely. This class of hydrogels are usually capable of self-assembling in to fibrous materials on its own (such as collagen and fibrin gels). The use of iron oxide/magnetic nanoparticles for a magnetic orientation approach is one of the most common method used to remote control the alignment of fibers during scaffold formation. Table 4 shows a summary of magnetic orientation fabrication methods to produce scaffolds for NTE.

**Figure 8:**
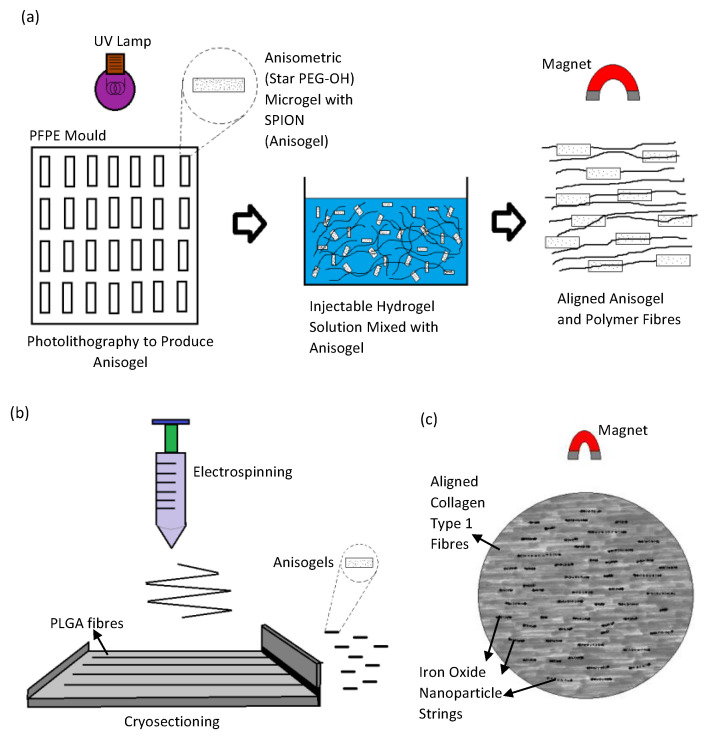
Magnetic orientation method. (**a**) The production of anisogels using a photolithography method. (**b**) Anisogels produced by using a electrospinning and cryo-sectioning. (**c**) Direct application of iron oxide nanoparticles into injectable collagen hydrogels.

**Figure 9:**
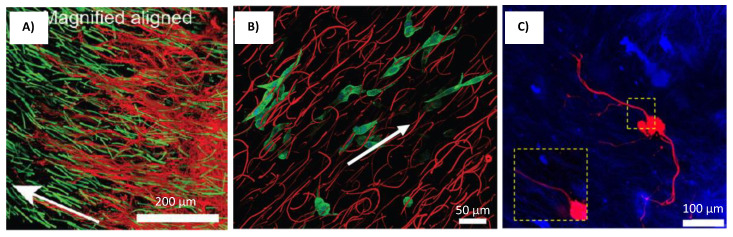
Cells cultured on different scaffolds produced using magnetic orientation. (**A**) DRGs (Red) were positioned in hydrogels with 3 vol% microgels (green) [26]. Reprinted with permission copyright © 2017 American Chemical Society. (**B**) Fibroblasts (stained in green) elongate in the direction of the oriented fibers (red) [27]. Reprinted with permission copyright © 2017 Wiley-VCH Verlag GmbH & Co. KGaA, Weinheim. (**C**) Confocal z stack image of a leech neuron grown in a magnetically aligned gel [28] Reprinted with permission copyright © 2016 American Chemical Society.

Research by Rose et al. reports the use of an injectable and magnetically controllable hydrogel that is capable of orientating nerve cells [26]. Fibrin fibers were aligned by magnetically orientated PEG anisogels [26]. The anisogel itself was made from six armed star shaped poly(ethylene oxide-star-propylene oxide) with acrylate end groups (star PEG-A), star-PEG-OH, and superparamagnetic iron oxide (SPION) using soft lithography technique (shown in Figure 8a) [26]. These rod shaped microgels would have mechano-physical properties that could push fibrin matrix to align according to the magnetic field direction [26]. The resulting injectable hydrogels were used to orientate chicken derived primary dorsal root ganglions (DRG) [26]. The cells orientation image in the scaffold is shown in Figure 9A. When it was magnetically aligned, the cells neurites orientated parallel to the axis of the microgels alignment [26]. The microgels itself is non adhesive to cells, and therefore, only guides the surrounding matrix in a singular direction [26]. These anisogels not only directs cellular proliferation but also subsequently guides the neurites axonal growth within the injectable hydrogel [26].

A similar approach was used by researchers Omidinia-Anarkoli et al., where short fibers containing SPION were fabricated to induce the aligned growth of DRG neurites [27]. However, the anisogel were fabricated with PLGA solution containing SPION using the electrospinning technique [27]. The fibers were then cut in to small tube-like structures with a cryo-sectioning device (shown in Figure 8b) [27]. These smaller fibers were then inserted into a fibrinogen and thrombin solution [27]. The magnetic field would then align these smaller fibers and subsequently lead to orientation of the self-assembling fibrin matrix [27]. The DRG neurons grown within the magnetically aligned scaffolds showed unidirectional proliferation along the anisogel orientation [27]. The cell orientation image in the scaffold is shown in Figure 9B. The fibrin hydrogels containing the anisogels, be it magnetically aligned or not, showed enhancement in its neurite development capabilities. This was mainly attributed to the cell adhesive properties to the anisogels [27]. However, the level of neuronal growth was far more prominent in the magnetically aligned hydrogels [27]. The neurons that proliferate along the anisogels exhibits impromptu electrical action from the calcium signaling process [27].

A study by Antman-Passig and Shefi shows a more direct application of iron oxide nanoparticles (IONPs) to direct fibers for neuronal alignment [28]. The iron oxide nanoparticles were directly inserted into the collagen hydrogel solution to align the collagen fibers during the gelling process (shown in Figure 8c) [28]. This is made possible by the formation of magnetic particle strings when a magnetic field is applied, which then pushes the assembling collagen fibers along the axis of the string’s alignment [28]. Leech neuronal cells cultured within the scaffolds showed orientated growth with little branching, which indicates exceptional neuronal pathfinding [28]. Around 60% of cells axons orientate within 15° of the magnetic strings and a total of 75% orientate within 30° of the angle of distribution. The cell orientation image in the scaffold is shown in Figure 9C. It was also reported that when a more dilute gel solution was used, the neurons grew along the particle strings instead of the resultant thinner collagen fibers [28]. Therefore, the orientation of neurites will occur closer in proximity to the magnetic strings if the collagen fibers could not sufficiently direct its proliferation [28].

The use of iron oxide nanoparticles, either directly or indirectly with the use of anisogels, have resulted in the subsequent orientation of self-assembling hydrogel fibers and subsequently, the neuronal cells cultured therein after a magnetic force is applied. The incorporation of other materials and nanoparticles allow a certain degree of control over how these fibers are assembled, and effectively unlocks different strategies for properly culturing neuronal cells. However, this technique involves only natural polymers that could self-assemble into microfibers, such as fibrinogen and collagen, which may limit the types of biomaterials applicable with this method.

### 4.5. Other Fabrication Methods for Producing Aligned Microstructures within Scaffolds

There have been other unique methods besides electrospinning, microfluidics, and magnetic orientation, to fabricate scaffolds with highly aligned internal structures for neuronal cell culture. Table 5 shows a summary of other unique fabrication methods to produce highly aligned scaffolds for NTE.

**Figure 10:**
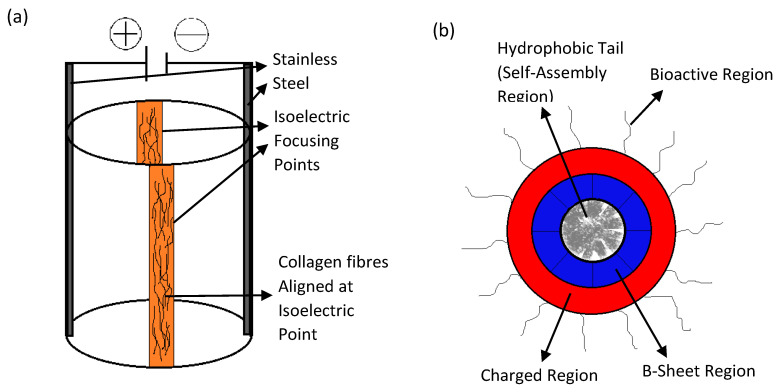
Novel methods for scaffold fabrication. (**a**) The iso-electric focusing procedure. (**b**) Cross-section of the self-assembling Tenacin C peptide.

**Figure 11:**
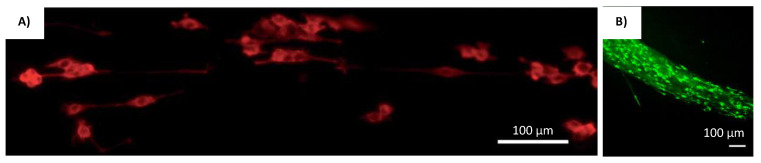
Cells cultured on scaffolds produced by isoelectric focusing and peptide self-assembly. (**A**) Representative fluorescent images of tubulin stained PC12 cells grown in isoelectric focused scaffolds [17]. Reprinted with permission copyright © Royal Society of Chemistry. (**B**) Flattened z-stack image of P19 embryonal carcinoma-derived neurons immunolabeled cells in 20% self-assembled tenascin B mimetic PA scaffolds [83]. Reprinted with permission copyright © 2016 Acta Materialia Inc. Elsevier Ltd.

Iso-electric focusing is a unique scaffold fabrication method utilized by researchers Abu-Rub et al. [17]. It involves the use of polytetrafluoroethylene (PTFE) tubes with two stainless steel plates running along its length, connected to a DC power source [17]. The pH gradient generated from the electrochemical reaction of the collagen solution therein creates an iso-electric point within the tube, where these fibers aggregate in an aligned manner as shown in Figure 10a [17]. The culture of embryonic rat neurites within these scaffolds reveals that it is capable of overcoming the inhibitory effect of myelin associated glycoprotein, which is a myelin degradation product at the site of neuronal injury [17]. This effect was not present when the DRG explants were cultured on poly-D-lysine substrates [17]. The cell orientation image in the scaffold is shown in Figure 11A. It was also reported here that iso-electric focused scaffolds only effects neurite alignment and not its length [17]. This may indicate that the orientation of cells in the injured site occurs along the axis of recovery from whence the scaffold could support the self-healing process.

Researchers Berns et al. also used a novel approach to fabricate neural scaffolds by utilizing biomimetic amphiphile (PA) derived from ECM glycoprotein Tenascin-C that could self-assemble into supramolecular nanofibers to guide the growth of neuron cells [83]. Standard solid phase peptide synthesis method, was used to synthesize PAs with FMOC-protected amino acids as shown in Figure 10b [83]. These were then coupled with rink-resin by shaking the resin in 30% Piperidine in N,N-dimethylformamide (DMF) [83]. The alignment of P19 embryonal carcinoma cultured within the neural scaffolds and its subsequent differentiation showed that the scaffold is capable of promoting neurite outgrowth [83]. The cell orientation image in the scaffold is shown in Figure 11B. Furthermore, these scaffolds enhanced the migration of neural progenitor cells, which makes it an excellent candidate as an injectable biomimetic gel capable of promoting self-healing [83].

Alignment of scaffolds internal microarchitecture has led to the improved performance of neural cell and the growth of its axons. Studying the configuration of natural tissue ECM’s structural protein has provided researchers a blue print on how certain cell types thrive. This orientation focused design is especially important to neuronal cells as it also directs their axonal extensions for proper tissue healing and restoration of functionality. Electrospinning has been the most used fabrication method in the field if NTE. Microfluidics and bioprinting has also been used frequently in recent years. The electrospinning and microfluidics method enables the creation of fibrous constructs using any type of biomaterials which greatly expand the possibilities to produce scaffolds in a cheaper and cost-effective manner. Bioprinting on the other hand could not produce these aligned scaffolds and would require the use of other fabrication methods to create an appropriate foundation for scaffold building. Despite this, bioprinting could still use its precise material and cell positioning to create microenvironments that enables axonal alignment. However, surgery is required to apply the scaffolds fabricated using these methods. This drove the search for injectable hydrogels and possible methods to remote control the configuration of their internal microstructures. Magnetic orientation method has been the most notable technique utilized for this purpose. Nevertheless, injectable hydrogels are usually made up of materials that could naturally self-assemble such as collagen and fibrin. This greatly limits the biomaterials applicable using this method. Hence, scaffold manufacturers need to consider all these fabrication technologies because some may excel in reducing the manufacturing cost and time while others may provide alternative treatment strategies.

## 5. Conclusions and Future Prospective

Development of scaffolds needs to follow the very specific delineation of tissue microarchitecture for efficient healing to take place at the site of injury. This includes taking into account, the mechanical integrity, orientation of internal components, and adherence properties of these tissue platforms. Biodegradation should also be considered, as the resultant tissues will replace the scaffolding. Neural tissues either from the brain, spinal cord, or peripheral nerves have a very specific, aligned internal structures that the cells therein take cues from to guide proliferation and axonal growth.

To produce scaffolds capable of properly culturing neuronal cells, specialized hydrogels, and fabrication techniques need to be utilized. In this review, we have discussed the many novel methods used to prepare scaffolds of neural tissue culture. Some of these fabrication techniques are considered evolution of a pre-existing technology that were greatly modified in order to produce specialized scaffolds for NTE applications. The bioprinting method could be seen as an application of the microfluidic method but with the precision of computer assisted design and production. With our current understanding of the methods mentioned here, we foresee an evolution of the electrospinning process into a more precise, computer assisted manner, whereby the application of fibers jetted out could be fine-tuned and molded as seen fit by the researchers. Chevron grooves could also be used in a bioprinting setup to produce multicomponent scaffolds with greater freedom in positioning these components when fabricating the structures. Isoelectric focusing could be utilized to induce internal microstructure or fiber alignment during the bioprinting process. These are just some ideas that could lead to innovative neural tissue scaffolds.

With the advent of injectable hydrogel technology, new novel methods have been developed which utilizes magnetic force and peptide self-assembly in order to achieve orientation of these internal ECM components in vivo. This class of hydrogels provides health care professionals with a non-surgery treatment that could promote the proper nerve restoration with much greater efficiency as well as the ability to instantly provide treatment on site. The magnetic/IONP method seem to be gaining popularity amongst the researchers working on injectable neural scaffolds. However, it could be more difficult to control the alignment of fibers or orientation of microstructures after injection. With a better understanding of magnetic fields, and more novel and effective technology of applying it, we may be able to create a more robust controllable 3D method of aligning these injectable hydrogels in situ. It could also be done in conjunction with the use of peptides to further improve alignment and functionality of the neural scaffolds. More novel soft materials developed for cell cultures could be utilized in this magnetic alignment methodology, to increase the repertoire of useable hydrogels.

Scaffold characterization is also another key factor to determine the improvements made to neural tissue culture and how it compares to other scaffolds fabricated with different methods. Therefore, quantification of neurite alignment should be standardized to make it easier to determine which methods are producing scaffolds that are yielding better neuronal alignment. As our understanding of tissue structure and cellular behavior increases, so does the innovations made to scaffold fabrication techniques, as discussed in this review. Therefore, new treatment strategies for neuronal diseases could be devised as our repertoire of scaffold fabrication methodology expands.

## Figures and Tables

**Table 1:** Summary of modified electrospinning methods used to produce NTE Scaffolds.

Method/Mechanism that Creates Fiber Alignment	Materials Used	Cells Used	Results/Advantages	Disadvantages	References
The orientated fibers were produced by applying high voltage power supply to blunt needles at two different locations	Poly-L-lactic acid (PLLA) fibrous scaffold	Human neural progenitor cell (HNPC)	- Greatly influences the migration pattern of HNPC - Enhances the potential for differentiation and nuclei polarization of the HNPCs	- Requires sugery for scaffold application - Mostly involve the use of synthetic materials due to its mechanical strength - If natural material is used, a sheath is necessary to hold its structure - Unable to include cells during the scaffold fabrication process due to the harsh environment involving high voltage and the presence of solvents	[64]
- Standard electrospinning setup with a rotating drum used as a ground collector - Hummer’s method was used to coat the scaffolds with GO	PLLA nanofibers-coated graphene oxide (GO)	- Schwann cells (SC) - Rat pheochromocytoma 12 (PC12) cells	- PLLA nanofibers improved growth of Schwann cells (SC) as well as directed its orientation in situ - This enhancements in differentiation capabilities is also exhibited by PC12 cells - GO improved cell attachment		[65]
Standard electrospinning setup with the addition of an aluminum foil-coated rotating mandrel to collect fibers jetting out of the blunt needle	Thermoplastic polycarbonate urethane (PCU) coated with Poly-l-Lysine (PLL) and Poly-l-Ornithine (PLO),	Dental pulp stem cells (DPSC)	- Highest speed of mandrel rotation (1800 RPM) produced the most aligned PCU fibers - With bioactive agents, dental pulp stem cells (DPSC) presented greater attachment and proliferation		[66]
Wet spinning process which includes a rotating collector water bath containing calcium chloride (CaCl2) and thrombin	Fibrin	Schwann cells	- Schwann cells showed remarkable orientation and attachment - Axonal regrowth was much faster in groups with the combined aligned electro spun fibrin scaffold/chitosan tube - Enhanced the morphogenesis of the cells as well as its motility function in situ		[70]
Wet spinning process which includes a rotating collector water bath containing calcium chloride (CaCl2) and thrombin	Fibrin	- Human mesenchymal stem cell (hMSC) - Dorsal root ganglions (DRGs) from postnatalSprague-Dawley rats - In Vivo studies—T9 Dorsal hemisection spinal cord injury of Sprague-Dawley rats	- Enhance the neuronal differentiation capabilities of hMSC and promote its outgrowth without the use of neurotrophic agents - Significant axonal outgrowth from the cells cultured therein		[69]
Two pole air gap electrospinning technique	Poly (ε-caprolactone-co-ethyl ethylene phosphate) (PCLEEP)	In Vivo studies—incised portion of adult female Sprague Dawley rats C5 spinal cord	- Highly aligned topographical cues - There were also greater cellular attachment potential and drug delivery control		[67]
Standard electrospinning set up with a conductive rotating collector with cellophane tape adhered horizontally and vertically onto copper wires of the collector	Poly(lactic-co-glycolic acid)(PLGA) and Poly Urethane (PU)	- PC12 cells - S42 cells	- PC12 cells showed greater proliferation on the aligned inner surface compared to the outer - Higher porosity of the inner layer, allowed greater cell adhesion		[12]
Standard electrospinning set with an L shaped sharp point needle to direct the polymer solution downward onto a rotating wooden disc	Polystyrene mixed with tetrahydrofuran (THF) and dimethylformamide (DMF)	Human astrocytoma cells (U373)	- The degree of alignment is improved as the fiber’s directionality gets more regulatedly aligned fibers provided better contact guidance		[68]

**Table 2:** Summary of modified microfluidic fabrication methods to produce scaffolds for NTE.

Method/Mechanism that Creates Fiber/Microstructure Alignment	Materials Used	Cells Used	Results/Advantages	Disadvantages	References
- Flow force from the microfluidic device was used to align the collagen fibers - Sodium alginate also acts as an insulator to fence the cellular proliferation	Core-Collagen Type 1 Sheath-Sodium Alginate	Cortical rod-shaped neural units (cortical units), Hippocampal rod-shaped neural units (hippocampal units), and mNSC rod-shaped neural units (mNSC units)	- Highly aligned neural tissue fibers were formed - Applicable to studies that involves the formation of spherical ends and how it connects with other rod-shaped units	- Requires surgery for scaffold application - In order to produce stronger fibers, its diameter is limited by the flow rate used during fabrication - Microfluidics chips need to be designed and fabricated first before the fibrous scaffolds could be produced - Clogging is a serious problem especially when multicomponent polymers are used	[71]
- Hydrostatic pressure aligns the ECM components of the Matrigel as well as the cells that are cultured therein - Architecture of the micropillars and channels enables controlled crosslinking of Matrigel which forms interchanging between zones of compact and sparse density	Matrigel	Rat cortical neurons prepared from a Sprague–Dawley embryonic rat	- The configuration of Matrigel crosslinking directs the growth of cells in the direction of medium flow		[72]
- Three microchambers for the core and sheath fluids are linked together by microchannels that has four chevron which differentiates the flow rate of core and sheath fluids - This mechanism maintains the fibers alignment after fluid injection	PCL (Polycaprolactone) and PEG (Polyethylene glycol)	Adult hippocampal progenitor cells (AHPCs) isolated from adult Fischer 344 rats	- This microfluidic mechanism maintains the fibers alignment after fluid injection		[73]
- The internal aligned multi-fibril architecture was created by the shear stress within the laminar flow, and the phase separating properties of the polymers used	Hydroxypropyl cellulose (HPC) and sodium alginate (Na-Alg)	- PC-12 Cells - DRG Cells were harvested from 10-week-old male Wistar rats - Human IPSC-derived dopaminergic neuron cells	- Guide neuron cells along the fibrils and permits the proliferation of its axon along the length of the fibers - Promotes differentiation of human IPSC-derived dopaminergic neuron cells into mature neuron cells		[74]
- Microfluidic chip was fabricated with PDMS using soft lithography technique - A wet spinning approach was used, where the fibers produced by the microfluidic chip is collected by a rotating spool in a water bath	Collagen Type 1 PEG	NG108-15 neuronal cell line	- Produced fibers that have high mechanical strength—Capable of directing the growth, migration, and axonal development NG108-15 cells		[75]

**Table 3:** Summary of bioprinting-based fabrication methods to produce scaffolds for NTE.

Method/Mechanism That Creates Fiber/Microstructure Alignment	Materials Used	Cells Used	Results/Advantages	Disadvantages	References
- Extrusion-based 3D bioprinting procedure - Clotting factor XIII increases the rate at which the bio-ink solidifies - Fibrin provides haptotactic cues that directs axonal alignment	- Fibrin, hyaluronic acid (HA), and polyvinyl alcohol (PVA)	Schwann cells	- The Schwann cell encapsulated within these scaffold shows dorsal root ganglion neurite growth along the longitudinal alignment of the fibers	- Requires surgery for scaffold application - Limited to the use of bioink that is viscous enough to hold its shape during fabrication - Involve a post-fabrication process that might be harmful to cells with in hydrogels - Increase in cost of production with the inclusion of 3D printing process - A very expensive method to produce scaffolds	[79]
- Digital light processing (DLP) based 3D printing - SDF-1 to promote neural stem cell NSCs migration into the microchannels - Microchannels guides axonal growth of the NSC	- polyethylene glycol diacrylate (PEGDA), methacrylated gelatine (Me-Gel), and methacylated hyaluronic acid (Me-HA)	Human neural stem/progenitor cells (NSCs)	- Microchannels with well-defined orientation shows axonal growth alignment of the NSCs along its length		[80]
- Electro spun scaffolds are used as a foundation for the scaffold - This scaffold was then coated with a mixture PEG (40%) and PEG-DA (60%) using 3D printing - 3D-coated fibers, enhanced NSC’s cellular proliferation and differentiation - PEG-DA enhances cellular attachment	- PEG (Polyethylene glycol) and PEG-DA-coated PCL (Polycaprolactone)/Gelatine	Neural stem cell (NSC)	- The alignment of NSC’s is most efficient in scaffolds with the PCL/Gelatine foundation - Produces highly aligned nanostructures with great mechanical stability		[81]
- 3D coaxial printing which uses a microfluidic approach was utilized for scaffold fabrication - NSC-34 cells were producing neurites that grows from the shell towards the core layer (with primary myoblast cells) - The two separate materials used promotes differentiation and growth of their respective cells	- Collagen methacrylate (ColMA) and gelatine methacrylate (GelMA)	NSC-34 (motor neuron-like cells), Primary myoblast cells	- Collagen and GelMA optimally promotes differentiation and growth of their respective cells - Dual hydrogel constructs could mimic actual tissue physiology		[82]

**Table 4:** Summary of magnetic orientation fabrication methods to produce scaffolds for NTE.

Method/Mechanism that Creates Fiber/Microstructure Alignment	Materials Used	Cells Used	Results/Advantages	Disadvantages	References
- The anisogel was made using soft lithography technique and mixed with fibrin gel - Neurites orientated parallel to the axis of the microgels alignment	- Fibrin, poly(ethylene oxide-star-propylene oxide) with acrylate end groups (star PEG-A), star-PEG-OH and superparamagnetic iron oxide (SPION)	- Chicken derived primary dorsal root ganglions (DRG)	- An injectable and magnetically controllable hydrogel	- Only hydrogels that could self-assemble are used in this fabrication method which limits the types of materials that could be utilized	[26]
- The anisogel was made using soft lithography technique and mixed with fibrin gel - Neurites orientated parallel to the axis of the microgels alignment	- Fibrin, PLGA solution containing SPION	- DRG neurons	- An injectable and magnetically controllable hydrogel - Neurons exhibits impromptu electrical action from calcium signalling process - Unidirectional proliferation along the anisogel orientation		[27]
- Direct addition of iron oxide nanoparticles in collagen gels - Neurons proliferated along the particle strings - Leech neuronal cells showed orientated growth with little branching	Collagen, Iron Oxide Nanoparticles	Leech Neuronal Cells, PC12 cells	- An injectable and magnetically controllable hydrogel - Little preparation necessary for efficient alignment		[28]

**Table 5:** Summary of other unique fabrication methods to produce highly aligned scaffolds for NTE.

Method/Mechanism that Creates Fiber/Microstructure Alignment	Materials Used	Cells Used	Results/Advantages	Disadvantages	References
- Isoelectric Focusing - Creates a pH gradient that aligns the collagen type 1 fibers - Cells align according to collagen type 1 fiber orientation	Collagen Type 1, polytetrafluoroethylene (PTFE) tubes with two stainless steel plates running along its length	- PC12 cells, - Embryonic - Dorsal Root Ganglia (DRGs) were dissected from E15 rat embryos	- DRG cells show the capability to overcome the inhibitory effect of myelin associated glycoprotein	- Requires surgery for scaffold application - Iso-electric focused scaffolds only effects neurite alignment and not its length	[17]
- Biomimetic amphiphile (PA) derived from ECM glycoprotein Tenascin-C that could self-assemble into supramolecular nanofibers and guide the growth of P19 embryonal carcinoma cells	biomimetic amphiphile (PA) derived from ECM glycoprotein Tenascin-C	P19 embryonal carcinoma cells	- Improves P19 cells viability and alignment - Enhance the migration of neural progenitor cells into the scaffolds		[83]
